# Structures of Human Transglutaminase 2: Finding Clues for Interference in Cross-linking Mediated Activity

**DOI:** 10.3390/ijms21062225

**Published:** 2020-03-23

**Authors:** Gi Eob Kim, Hyun Ho Park

**Affiliations:** College of Pharmacy, Chung-Ang University, Seoul 06974, Korea; gieob1994@naver.com

**Keywords:** transglutaminase 2, structure-based drug design, protein structure, peptide mimetic

## Abstract

Human transglutaminase 2 (TGase2) has various functions, including roles in various cellular processes such as apoptosis, development, differentiation, wound healing, and angiogenesis, and is linked to many diseases such as cancer. Although TGase2 has been considered an optimized drug target for the treatment of cancer, fibrosis, and neurodegenerative disorders, it has been difficult to generate TGase2-targeted drugs for clinical use because of the relatively flat and broad active site on TGase2. To design more specific and powerful inhibitors, detailed structural information about TGase2 complexed with various effector and inhibitor molecules is required. In this review, we summarized the current structural studies on TGase2, which will aid in designing drugs that can overcome the aforementioned limitations.

## 1. Introduction

Transglutaminases (TGases) are enzymes that catalyze the cross-linking reaction between the γ-carboxamide of a glutamine residue and the ε-amino group of a lysine residue; they also catalyze the deamidation reaction of glutamine residues to generate glutamic acid in the presence of water [1,2]. These enzymes exhibit cross-linking activity, which helps create stable, barrier-like structures such as blood-clots, skin, and hair [1,3,4]. In humans, eight isoforms of TGases, TGase1~7 and Factor XIII, have been identified and characterized [5].

Human TGase2 (also known as tissue TGase) is a multi-functional, ubiquitous enzyme that is important for various cellular processes, including apoptosis [6,7,8], development [9,10], differentiation [11,12], wound healing [13,14], and angiogenesis [14,15]. Human TGase2 comprises 687 amino acids (~70 kDa) and has a four-domain organization. It contains the N-terminal β-sandwich domain, the catalytic core domain containing the catalytic triad, and two β-barrel domains (β-barrel1 and β-barrel2) [16]. Using this domain organization, TGase2 performs multiple functions; it acts as a GTPase [17,18,19], kinase [20,21], protein disulfide isomerase [22], and scaffolding factor [23], and performs the traditional activity of cross-linking by transamidation. The multiple functions of TGase2 are controlled in various ways in the cell, such as by cellular cofactors [24,25], spatial localization of TGase2 [13,26], endogenous amine compounds and proteins [27,28,29,30,31], and post-translational modifications [32]. Non-functional and dysregulated TGase2 is related to various human diseases, such as celiac disease [33], inflammatory disease [34], many types of cancers [3,35,36,37,38], tissue fibrosis [39], diabetes [40], pulmonary hypertension by fibrogenic remodeling [41], and neurodegenerative diseases [42,43,44]. It has been reported that overexpression or over-activity due to mutations in TGase2 leads to celiac disease [45], inflammatory disease [34], and tissue fibrosis [39]. Both downregulated and upregulated TGase2 are known to cause many types of cancer [8,46]. TGase2 has been found to be downregulated in sarcoma [35,47], whereas its overactivation has been observed in pancreatic and breast cancer [46,48]. Hyperactivity of TGase2 in the pathogenic stages of neurodegenerative diseases, including Alzheimer’s disease (AD), has also been reported [49,50,51,52]. These studies show that hyperactivated TGase2 cross-links several AD-related proteins, including tau, Aβ, and α-synuclein, causing the accumulation of these proteins in patients with AD [50,51,52]. Misregulated TGase2 activity due to mutations has been found in the early stages of type 2 diabetes, indicating that its activity is also linked to metabolic diseases [40].

Owing to its biologically and pathogenically important functions, TGase2 has been considered a major therapeutic target for the treatment of many human diseases caused by dysregulated TGase2 [29,53,54,55,56]. The aim of this review was to summarize the recent progress in the structural studies of TGase2, which can aid in the development of drugs targeting this enzyme. We will discuss its structural complexity and several TGase2 structures complexed with effector molecules.

## 2. Multi-functional TGase2

**Figure 1:**
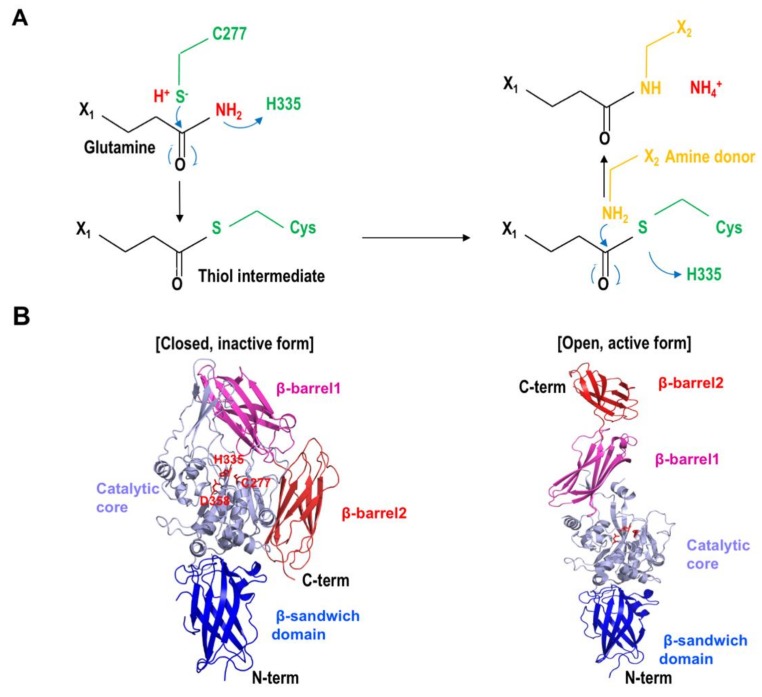
Reaction mechanism of transglutaminase 2 (TGase2). (**A**) Transamidation reaction mechanism of TGase2. Residues in green are from TGase2. Two consecutive nucleophilic attacks by the thiol group from the active site on TGase2 and the amine donor are shown. (**B**) Figures of the closed inactive form and the open active form of TGase2 showing the catalytic triad composed of C277, H335, and D358. The four distinct domains, β-sandwich domain, catalytic core domain, β-barrel1, and β-barrel2, are visually distinguished by the different colors.

TGase2 is a multi-functional protein. Cross-linking, deamination, and amine incorporation activities are the main functions of TGase2, GTPase, disulfide isomerase, and kinase. Scaffolding activities are also reported as minor functions of TGase2 [16,57]. The principal transamidation activity is performed by the catalytic triad, composed of C277, H335, and D358, on the active site of TGase2. The thiol functional group of C277 on TGase2 works as a nucleophile and attacks the carboxamide of a glutamine residue, forming a thioester intermediate during the transamidation reaction. Then, this unstable intermediate is broken down by the ε-amino group of the lysine residue, producing a stable isopeptide bond that cross-links the two substrates (Figure 1A).

The protein disulfide isomerase (PDI) activity of TGase2, which was initially reported in studies of RNase A [22] and adenine nucleotide translocator 1 (ANT1) [58], is another important function of TGase2. An initial PDI activity study showed that reduced, denatured, inactive RNase A was converted into the renatured, active form by the PDI activity of TGase2 [58]. Neither Ca^2+^ (activator of transamidase activity) nor guanosine triphosphate (GTP, inhibitor of transamidase activity) affect the PDI activity of TGase2 [58]. A mutagenesis study showed that the cysteine residue in the active site is not critical for the PDI activity of TGase2, indicating that the two reactions, transamidation and PDI, are independent. Another TGase2 substrate for PDI activity is the ANT1 protein, which is one of the components of the mitochondrial membrane permeability transition pore (PTP) [59,60]. During apoptosis, ANT1 is oligomerized and forms a pore on the mitochondrial membrane owing to the cross-linking and PDI activities of TGase2 [58]. This causes the cell to release various pro-apoptotic molecules, leading to apoptosis. This indicates that the PDI activity of TGase2 is involved in mitochondria-dependent apoptosis.

When TGase2 was first characterized, it was assumed that the nucleotide-binding pocket on TGase2 strongly indicated that it might have GTPase and kinase activities. Since then, GTPase activity during signal transduction has been reported [17]. In this study, TGase2 mediated between the α1-adrenergic receptor and phospholipase Cδ in the liver during proliferation signaling of hepatocytes, which is critical for the GTPase activity of TGase2 [17]. A study on the kinase activity of TGase2 on insulin-like growth factor-binding protein-3 (IGFBP-3) was the first to report the kinase activity of TGase2 [61]. The study indicated that TGase2 phosphorylated both IGFBP-3 and IGFBP-5 at multiple serine residues. The kinase activity of TGase2 was inhibited by Ca^2+^, which is known to be an activator of the transamidation activity of TGase2 [61]. Many additional substrates, including P53 [21], histones [20], retinoblastoma protein (Rb) [62], the transmembrane protein CDH1 [63], and the extracellular protein MMP-3 [63], have since been reported. TGase2 can form a complex with fibronectin and collagen in the extracellular matrix (ECM) and mediate the interaction between fibronectin and integrins during the cell adhesion process. Its scaffolding activity, which is not related to the cross-linking activity for ECM turnover, has also been highlighted [23,64].

As the functions of TGase2 are critical for the survival of the organism owing to its involvement in various cellular processes, TGase2 activities have to be finely controlled. It has been reported that the activity of TGase2 is controlled by cellular cofactors, spatial localization, endogenous protein regulators, and post-translational modifications [13,24,25,26,27,28,29,30,31,32]. Various cellular ions and nucleotides are the main regulators of the multi-functional activities of TGase2. Although the activation mechanism is still unclear, the most well-known activator of the transamidation activity of TGase2 is the Ca^2+^ ion. Ca^2+^ was initially assumed to promote the structural transition of TGase2 to its active form. A recent study, however, showed that Ca^2+^ binding to TGase2 did not lead to a dramatic structural transition [65]. In contrast, nucleotides such as GTP, GDP (guanosine diphosphate), and ATP (adenosine triphosphate) are well-known inhibitors of the transamidation activity of TGase2 [66]. Nucleotide binding to the relatively well-defined nucleotide-binding site on TGase2, which is located between the catalytic core domain and the first β-barrel, maintains the compact, inactive form of TGase2 via the formation of hydrogen bonds between a cysteine in the active site and a tyrosine residue nearby; this blocks the structural transition to the active form [67,68,69].

Endogenous amine compounds, such as cystamine, cysteamine, spermidine, and histamine, are another class of TGase2 inhibitors [53,70]. As these amine compounds can work as amine donors that can cross-link with glutamine in the substrate, they can inhibit the transamidase activity of TGase2 by competing with natural substrates [28]. Another known inhibition mechanism used by the amine compounds is the direct modification of the active site cysteine (C277) and other surface-exposed cysteines on TGase2, which are crucial for TGase2 activity, by the formation of a disulfide bond [71,72]. It has been reported that several disulfide bonds on TGase2, which are formed or unformed based on the oxidation/reduction states, were critical for the regulation of allosteric activity. This indicates that the amine compound-mediated cysteine modification and the formation of disulfide bonds are important processes for TGase2 activity control [31,73]. Endogenous proteins can also control TGase2 activity. Ribosomal proteins RPL7a and RPL13, which are the most well-known endogenous protein inhibitors of TGase2, inhibit transamidase activity by binding to the β-barrel2 domain of TGase2 [29]. Besides the direct activity control by endogenous small molecules, macromolecules, and ions, cytokine- and reactive oxygen species (ROS)-mediated indirect mechanisms of TGase2 activity control have also been suggested [30,74,75].

## 3. Structures of TGase2 Complexed with Various Effectors

**Figure 2:**
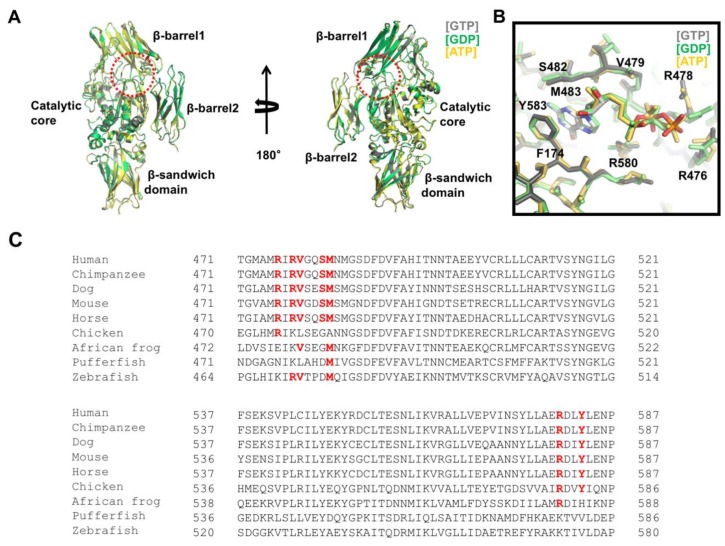
Structural analysis of the nucleotide-binding site on TGase2. (**A**) Structural comparison of TGase2 complexed with different nucleotides. Three different structures were superimposed and compared. The dotted red circle indicates the nucleotide-binding site located in between the catalytic core domain and the β-barrel1 domain. (**B**) Close-up view of the nucleotide-binding site on TGase2. Residues involved in the interaction with the nucleotides are shown. (**C**) Sequence comparison of TGase2 in various species. Residues important for binding with nucleotides are shown in red bold font.

The multi-functional activity of TGase2 is finely controlled by various molecules in the cell. To understand the molecular mechanism of the activity control of TGase2 by various effector molecules, structural studies of TGase2 complexed with effector molecules have been performed. The structures of three nucleotides, GTP, GDP, and ATP-binding TGase2 have been reported [67,69,76]. These structures showed that a unique nucleotide-binding site was present in between the catalytic core and the first β–barrel (Figure 2A). The three structures were nearly identical, having a root mean square deviation of ~0.5 Å with each other, indicating that different nucleotides did not affect the overall structure of TGase2 (Figure 2A).

Although TGase2 possesses GTPase and ATPase activities, the major role of the nucleotides is to inactivate its activity by blocking structural transition, which is important for retaining the activity of TGase2. Structural analysis of the GTP-binding site of TGase2 indicated that three positively charged arginines, R476, R478, and R580, surround the negatively charged phosphate moieties of the nucleotide by forming hydrogen bonds, whereas hydrophobic residues from TGase2, including F174, V479, and M483, stabilize the hydrophobic base moiety on the nucleotides via hydrophobic interactions (Figure 2B) [67]. Among the residues accommodating the nucleotides, R580 was the most critical residue for the nucleotide interaction. Mutations in R580, which formed two hydrogen bonds with the phosphate of the nucleotides, abolished the nucleotide-binding capacity and eliminated its sensitivity to GTP [77,78]. R478 is involved in nucleotide binding via the formation of hydrogen bonds with the γ-phosphate of GTP. The structure of GDP-bound TGase2 showed that the side chain of R478 moved toward the γ-phosphate position, which is the most critical structural difference between GTP-bound and GDP-bound TGase2 (Figure 2B) [69]. Binding preference between GTP and ATP on TGase2 was identified by a structural study [67]. S482 and Y583 residues are involved in the interaction with only GTP, not ATP, indicating that GTP is the preferable nucleotide owing to a tighter interaction. Indeed, TGase2 activity is more strongly inhibited by GTP than ATP [25]. For nucleotide binding, Mg^2+^ is not necessary, unlike other GTP-binding proteins, including various small G proteins [67].

Amino acid sequence analysis of TGase2 indicates that the nucleotide-binding pocket generated by the residues R476, R478, V479, S482, M483, R580, and Y583 is only conserved in mammals (Figure 2C). This site is not formed in birds, frogs, or fish, indicating that the use of nucleotides in TGase2 has evolved over a long period of time.

## 4. Structures of TGase2 Complexed with Inhibitors Derived from Peptide Mimetics

**Figure 3:**
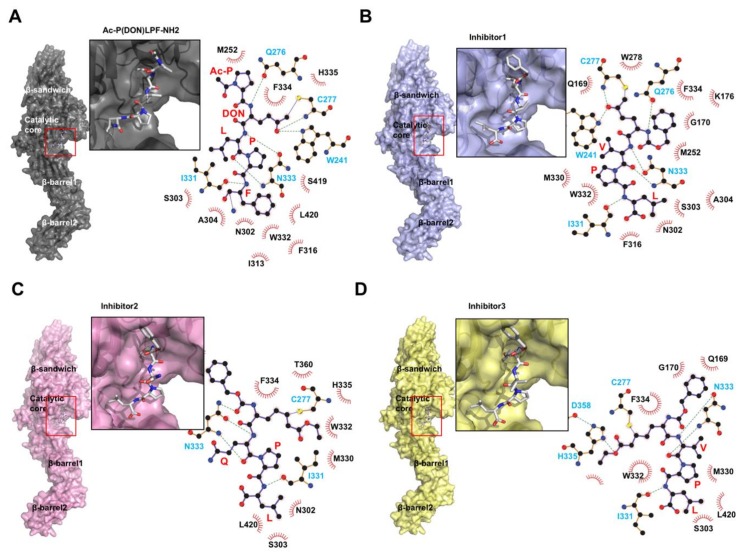
Structural analysis of TGase2 complexed with peptide mimetic inhibitors. Structure of TGase2 complexed with peptide mimetic, Ac-P(DON)LPF-NH2 (**A**), with peptide inhibitor1, N-[(benzyloxy)carbonyl]-6-diazonio-5-oxo-L-norleucyl-L-valyl-L-prolyl-L-leucine (**B**), with peptide inhibitor2, N~2~-[(2S)-2-{[(benzyloxy)carbonyl]amino}-7-ethoxy-7-oxoheptanoyl]-L-glutaminyl-L-prolyl-L-leucine (**C**), and with peptide inhibitor3, N-[(2R)-2-{[(benzyloxy)carbonyl]amino}-7-ethoxy-7-oxoheptanoyl]-L-valyl-L-prolyl-L-leucine (**D**). The red box indicates the inhibitor-binding site. The magnified figure of the inhibitor in the inhibitor-binding site of TGase2 is provided. The binding details, generated by LIGPLOT+ [80], are shown at the right side of each panel. The black and cyan residues indicate the hydrophobic interaction and hydrogen bond, respectively.

While nucleotides maintain the closed form and inhibit TGase2 activities, substrate derived-peptide mimetics, which work as irreversible inhibitors, promote the open, active conformation of TGase2 (Figure 1B and Figure 3A) [79]. The first introduced inhibitor peptide, known as Ac-P(DON)LPF-NH2, where DON is the electrophilic amino acid 6-diazo-5-oxo-L-norleucine, derived from the penta-peptide PQLPY from gluten, is one of the substrates of TGase2. The TGase2/Ac-P(DON)LPF-NH2 complex structure shows that the peptide mimetic attaches to the active site C277 via a thioether covalent linkage (Figure 3A) [79]. The LPF peptide region of the inhibitor is well-positioned toward the hydrophobic pocket formed by S303, A304, I313, F316, I331, and L420 on TGase2. W241, Q276, C277, and N333 form hydrogen bonds with the inhibitor. Since this inhibitor-bound, open form structure was introduced, three more TGase2 structures complexed with peptide mimetic inhibitors have been deposited in The Protein Data Bank without publication. All three peptide mimetic inhibitors are modified versions of Ac-P(DON)LPF-NH2. The first inhibitor is N-[(benzyloxy)carbonyl]-6-diazonio-5-oxo-L-norleucyl-L-valyl-L-prolyl-L-leucine. This inhibitor is also a penta-peptide mimetic containing a VPL sequence instead of an LPF sequence. Similar to Ac-P(DON)LPF-NH2 binding, this inhibitor also irreversibly binds to C277 on TGase2 via a thioether linkage (Figure 3B). Hydrogen bonds are formed between the inhibitor ketone and the indole of W241, and the backbone amide of C277. The peptide-binding pocket of the other three inhibitors, formed by S303, A304, I313, F316, I331, and W332, is also similar to the Ac-P(DON)LPF-NH2 binding pocket. The second peptide mimetic inhibitor is N~2~-[(2S)-2-{[(benzyloxy)carbonyl]amino}-7-ethoxy-7-oxoheptanoyl]-L-glutaminyl-L-prolyl-L-leucine. This inhibitor contains a QPL sequence. QPL binds to the conserved substrate- or peptide mimetic inhibitor-binding pocket. Although this peptide mimetic also covalently binds to C277 on TGase2, which is similar to that observed in Ac-P(DON)LPF-NH2 binding, because of the absence of indole, the peptide inhibitor cannot form hydrogen bonds with C277 and the neighboring W241 (Figure 3C). Instead, N333 is more tightly involved in the peptide binding, forming three hydrogen bonds (Figure 3C). The last peptide mimetic inhibitor is N-[(2R)-2-{[(benzyloxy)carbonyl]amino}-7-ethoxy-7-oxoheptanoyl]-L-valyl-L-prolyl-L-leucine. This peptide contains a VPL sequence, and its interaction is similar to that of the second inhibitor. The modified, long side chain at the second position of this penta-peptide inhibitor interacts with C277 via a thioether linkage, and with H335 and D358 via hydrogen bonds in the deep active site (Figure 3D). This is the only peptide inhibitor that can interact with all three residues from the catalytic triad in the active site of TGase2.

## 5. Future Perspectives

With important roles in many pathological conditions and various activities, TGase2 has been considered an optimized target for therapeutic intervention [53,54,81]. In this effort, TGase2-targeting drugs have developed. Although several small molecules and peptide mimetic inhibitors have shown inhibiting capacity in animal models and clinical trials, no drugs are currently in use in clinical settings.

The screening and discovery of TGase2-targeting drugs by examining small molecules was not initially successful. Owing to its relatively flat and broad active site, finding small molecules that target the active site of TGase2 has always been challenging. To overcome this issue, many researchers have used peptide mimetics to cover the broad active site. Because of this effort, several potential peptide mimetic inhibitors are currently in clinical trials [82]. The nucleotide-binding site on TGase2 has also been targeted by non-hydrolyzable GTP analogs, which can bind to and maintain the inactive (closed) form of TGase2. However, this strategy was not successful owing to specificity [83]. To design high affinity optimized inhibitors more efficiently, structural information is critical and has been employed (Table 1).

**Figure 4:**
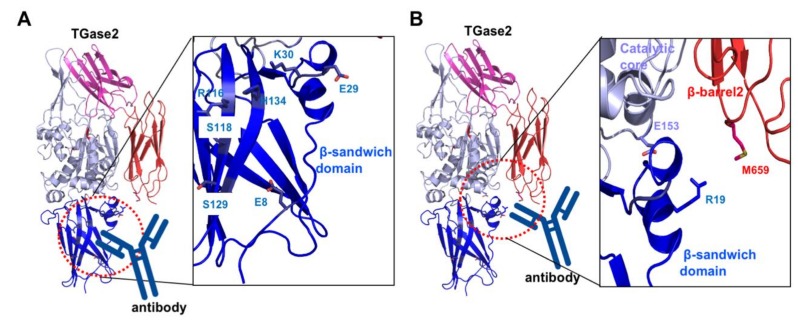
Structural analysis of celiac autoantibody-binding sites, site 1 (**A**) and site 2 (**B**). Close-up view of the autoantibody-binding sites (right panel). Residues that are involved in the interaction with the autoantibody are shown.

Besides finding small molecules and peptide mimetics that target the active site or the nucleotide-binding site of TGase2 for pharmaceutical purposes, various approaches to discover drugs for TGase2 inhibition are undergoing. One of the representative cases is the search for an antibody against TGase2. Celiac disease is caused by an autoimmune response to dietary gluten antigens. This antigen produces autoantibodies against TGase2 [84]. The effect of autoantibody in the fibroblasts from celiac disease patient [85], and B cell tolerance and autoantibody production in mouse models have been studied recently [86]. Recent structural and biophysical studies mapped the autoantibody-binding sites to TGase2 [87]. The first site is located in the N-terminal β-sandwich domain. Residues E8, E29, K30, R116, S118, S129, and H134 are involved in the antibody binding (Figure 4A) [87]. The second autoantibody-binding site is the cleft region formed by the β-sandwich, catalytic core, and β-barrel2 domains (Figure 4B) [88]. R19, E153, and M659 are the residues responsible for binding to the autoantibodies [88]. The inhibition of autoantibody interactions with TGase2 might be a good strategy for the treatment of celiac disease. The investigation of small interfering RNA (siRNA) targeting TGase2 expression has also been attempted for the development of drugs for the treatment of several types of cancers related to the aberrant expression of TGase2 [89]. Endogenous TGase2 inhibitors, such as the ribosomal proteins RPL7a and RPL13 might be important sources for TGase2 targeting [29]. Structural studies of TGase2 with various endogenous inhibitors need to be performed to aid drug design.

## Figures and Tables

**Table 1:** Structures of TGase2 complexed with effectors and inhibitors.

Effectors/Inhibitors	Closed/Open	PDB	References
GDP	Closed	1KV3	[76]
GTP	Closed	4PYG	[67]
ATP	Closed	3LY6	[69]
Ac-P(DON)LPF-NH2	Open	2Q3Z	[79]
Inhibitor1	Open	3S3J	N.A.
Inhibitor2	Open	3S3P	N.A.
Inhibitor3	Open	3S3S	N.A.

PDB, The Protein Data Bank; GDP, guanosine diphosphate; GTP, guanosine triphosphate; N.A., Non-Available.
